# Machine Learning for Medical Image Translation: A Systematic Review

**DOI:** 10.3390/bioengineering10091078

**Published:** 2023-09-12

**Authors:** Jake McNaughton, Justin Fernandez, Samantha Holdsworth, Benjamin Chong, Vickie Shim, Alan Wang

**Affiliations:** 1 Auckland Bioengineering Institute, University of Auckland, 6/70 Symonds Street, Auckland 1010, New Zealand; jmcn735@aucklanduni.ac.nz (J.M.);; 2 Department of Engineering Science and Biomedical Engineering, University of Auckland, 3/70 Symonds Street, Auckland 1010, New Zealand; 3 Faculty of Medical and Health Sciences, University of Auckland, 85 Park Road, Auckland 1023, New Zealand; 4 Centre for Brain Research, University of Auckland, 85 Park Road, Auckland 1023, New Zealand; 5 Mātai Medical Research Institute, 400 Childers Road, Tairāwhiti Gisborne 4010, New Zealand

**Keywords:** cross-modality synthesis, CT to MRI, modality translation, medical image generation, deep learning, medical imaging

## Abstract

Background: CT scans are often the first and only form of brain imaging that is performed to inform treatment plans for neurological patients due to its time- and cost-effective nature. However, MR images give a more detailed picture of tissue structure and characteristics and are more likely to pick up abnormalities and lesions. The purpose of this paper is to review studies which use deep learning methods to generate synthetic medical images of modalities such as MRI and CT. Methods: A literature search was performed in March 2023, and relevant articles were selected and analyzed. The year of publication, dataset size, input modality, synthesized modality, deep learning architecture, motivations, and evaluation methods were analyzed. Results: A total of 103 studies were included in this review, all of which were published since 2017. Of these, 74% of studies investigated MRI to CT synthesis, and the remaining studies investigated CT to MRI, Cross MRI, PET to CT, and MRI to PET. Additionally, 58% of studies were motivated by synthesizing CT scans from MRI to perform MRI-only radiation therapy. Other motivations included synthesizing scans to aid diagnosis and completing datasets by synthesizing missing scans. Conclusions: Considerably more research has been carried out on MRI to CT synthesis, despite CT to MRI synthesis yielding specific benefits. A limitation on medical image synthesis is that medical datasets, especially paired datasets of different modalities, are lacking in size and availability; it is therefore recommended that a global consortium be developed to obtain and make available more datasets for use. Finally, it is recommended that work be carried out to establish all uses of the synthesis of medical scans in clinical practice and discover which evaluation methods are suitable for assessing the synthesized images for these needs.

## 1. Introduction

Medical imaging is a routine part of the diagnosis and treatment of a variety of medical conditions. Due to limitations, including the acquisition time of imaging methods and the cost of obtaining medical images, patients may not receive all the imaging modalities that they could benefit from. A possible solution to this is to use deep learning methods to generate synthetic medical images which estimate these modalities from scans the patient did receive.

For example, the diagnosis of brain disorders is often informed by brain scans obtained from the patient. The purpose of such neuroimaging is to rule out or diagnose a variety of conditions caused by lesions in the central nervous system. The most widely used imaging modalities for this purpose are magnetic resonance imaging (MRI) and computerized tomography (CT). MRI is much more sensitive to conditions such as stroke, offering better contrast of soft tissues and excellent anatomical detail in comparison to CT scans; however, MRIs tend to take longer, and be less available and more expensive [1]. MRI is also not appropriate for patients with metal implants or claustrophobia. Due to these limitations, CT scans tend to be the first and often only scan a patient receives. Furthermore, compared to CT scans, MRIs provide a more accurate registration to most commonly used brain atlases. Benefitting from the advantages provided by MRI by synthesizing an MRI from a patient’s CT scan would therefore improve the treatment of patients presenting with brain disorders.

Deep learning can be utilized to generate images and therefore be applied to this problem. A limitation in using deep learning for medical imaging tasks is the availability of large datasets, a distinguishing factor in terms of what types of deep learning frameworks are suitable. Two commonly used frameworks for image synthesis are generative adversarial networks (GANs) and convolutional neural networks (CNNs). A GAN is a framework that consists of two models—a generator and discriminator—which are simultaneously trained [2]. The generator captures the data distribution of the training data and attempts to generate data which fits within this distribution, whilst the discriminator is presented with one piece of data and estimates whether it was generated by the generator. The generator and discriminator then engage in a two-player game, trying to become better at their respective tasks. A CNN is a framework that processes pixel data, and which is often used to detect objects in images [3]. In a medical context, one of the most widely used CNNs is U-Net, which is most commonly used for segmentation tasks [4].

A variety of evaluation metrics are used to assess the performance of deep learning models for medical image synthesis. Many of the metrics used to assess the performance of medical image synthesis models are the same as those used in general image synthesis tasks. Metrics assess the difference between two images—the one generated by the model and the ground truth image. Commonly used metrics include the mean error (ME), mean absolute error (MAE), and mean squared error (MSE) which compare pixel intensities.

The purpose of this study is to review the work that has been carried out on medical image synthesis. In medical settings, there is a shortage of large datasets suitable for supervised learning, so this review will consider studies which use supervised learning, unsupervised learning, or both.

## 2. Methodology

### 2.1. Search Strategy

This search was completed using the Preferred Reporting Items for Systematic Reviews and Meta-Analyses (PRISMA) guidelines. The focus was establishing what work had been carried out in terms of developing machine learning models which can translate medical images into different modalities. Therefore, the machine learning frameworks used and dataset details, including body parts studied and modalities studied, were variables of interest. Articles were included for this review if they conducted original research using machine learning methods to translate medical images from one modality into a different modality. Keywords were developed in three categories—machine learning, image generation, and medical imaging—to address these criteria. The keywords in each category are shown in Table 1.

### 2.2. Screening Process

Articles published in journals up until July 2023 (inclusive) were searched using PubMed. Additionally, relevant preprints were identified using ArXiv. Search queries were developed by combining keywords from the same category with the OR operator and combining the categories using the AND operator. The first screening phase involved screening articles based on their title and abstract to remove articles which were not relevant to the scope of the review or due to the exclusion criteria. In the final screening phase, papers were assessed based on the full text. Included papers then underwent data extraction. Reasons for exclusion included papers which were not written in English; were not able to be accessed; did not comprehensively describe an original study; were theses, reviews, or notes; did not give sufficient details on the dataset used to train the model; focused on reconstructing medical images to improve resolution; or focused on translating images into the same modality but with different acquisition parameters.

### 2.3. Data Extraction

From the included papers, the title, author details, year of publication, dataset size, part of the body the dataset contained, input modality, output modality, motivations stated for medical image synthesis, machine learning methods, and evaluation methods were extracted. Categories were developed to allow the included papers to be grouped based on the extracted data, and the categories are described in Table 2. Data extraction was performed on two separate occasions and compared to decrease the chance of human error. Studies in the same article were counted separately if they used different datasets for training or synthesized different modalities.

## 3. Results

**Figure 1:**
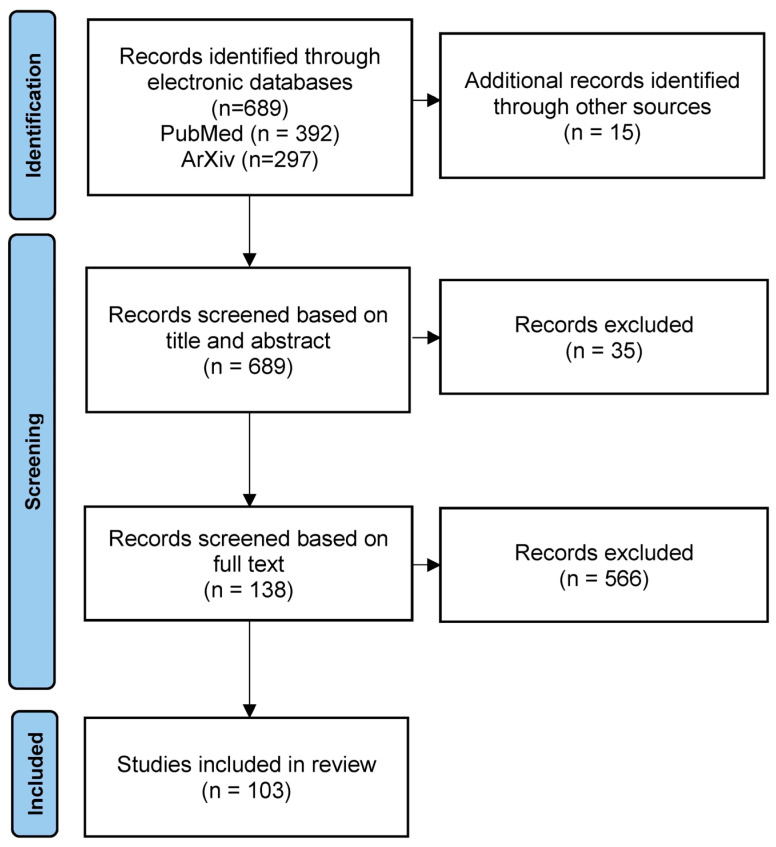
The PRISMA diagram detailing this systematic review.

Figure 1 shows the PRISMA flowchart for this review. A total of 392 articles were identified from PubMed, and 297 articles were identified from ArXiv. A further 15 articles which had already been identified as relevant were included from various sources. After title and abstract screening, 138 papers remained, and after screening of the full text, 99 articles were included, which documented 103 studies (Table 3).

### 3.1. Modalities Synthesized

**Figure 2:**
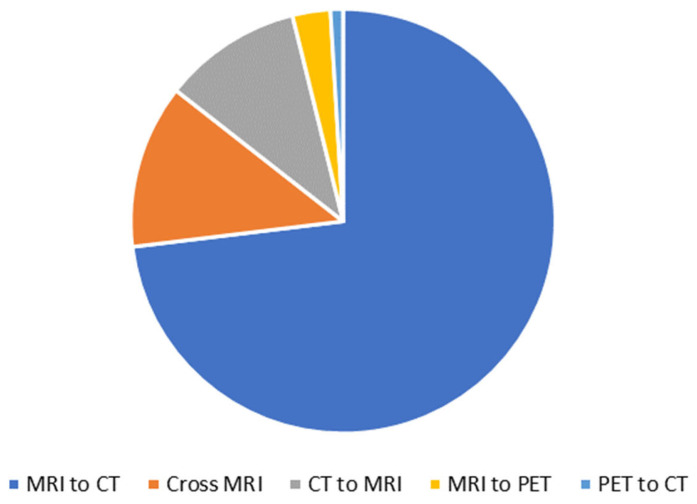
Breakdown of type of synthesis.

Figure 2 shows the breakdown of the types of synthesis in the included studies. Most studies (76) investigated MRI to CT synthesis, with the majority of these being motivated by MRI-only radiation therapy. Thirteen studies investigated Cross-MRI synthesis, which included T1 to T2 and T2 to FLAIR; often, these studies used a dataset with more than two MRI modalities and performed synthesis between many of the different modalities. All Cross-MRI synthesis studies used datasets of the brain. Eleven of the studies investigated CT to MRI synthesis, three studies investigated MRI to PET synthesis, and one study investigated PET to CT synthesis.

### 3.2. Year of Publication

**Figure 3:**
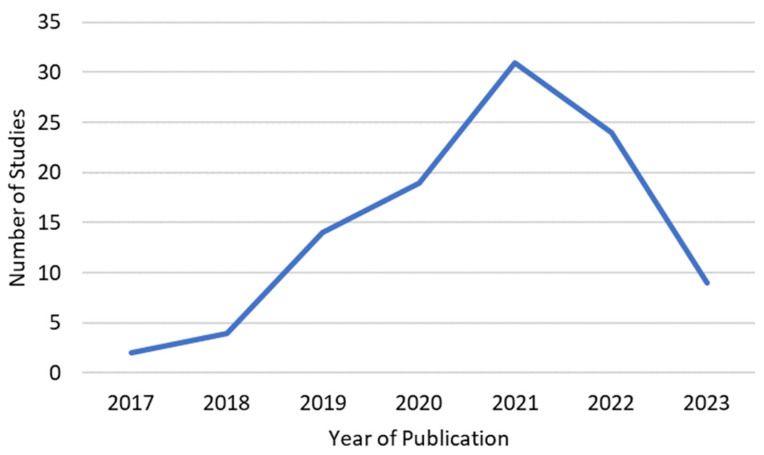
Year of publication of the reviewed studies.

Although no restriction was placed on the year of publication in the literature search, all included papers were published since 2017 (Figure 3). Between 2017 and 2021, the number of papers published appears to grow exponentially, with a drop from 31 studies in 2021 to 24 studies in 2022. Nine studies were from 2023, however, the literature search only included papers until July 2023.

### 3.3. Evaluation

**Figure 4:**
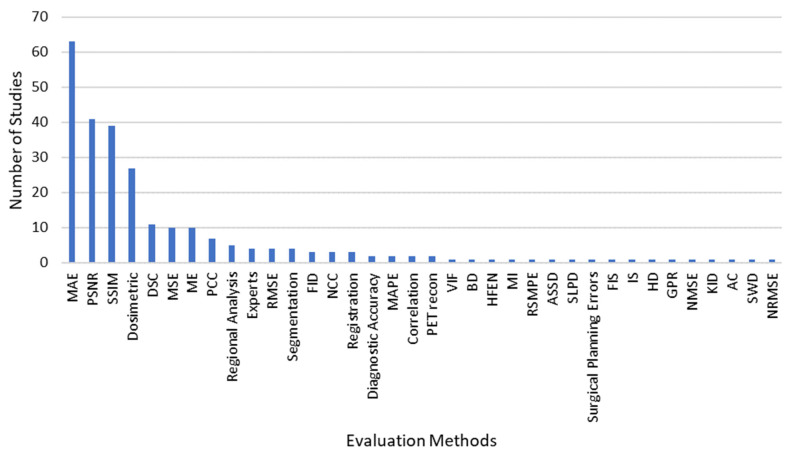
Methods for evaluating the synthetic images.

A total of 36 different methods were used to evaluate model performance (Figure 4). MAE (mean absolute error), PSNR (peak signal-to-noise ratio), and SSIM (structural similarity index) were the three most used evaluation metrics. It was common for studies motivated by MRI-only radiation therapy to use dosimetric evaluation; this was present in 27 studies. Dosimetric evaluation compared the radiation dosage plan based off the synthetic CT to that which the patient received based on the true CT.

### 3.4. Motivations

**Figure 5:**
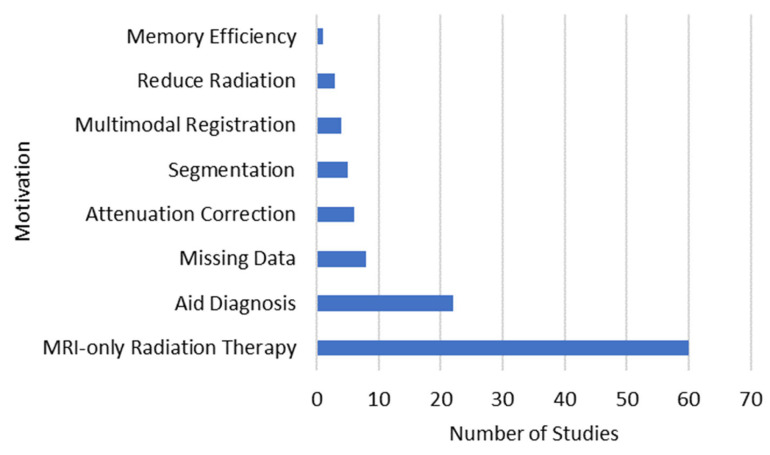
Stated motivations for medical image synthesis.

There were multiple motivations mentioned across the surveyed studies (Figure 5). The most common motivation was to achieve MRI-only radiation therapy, which was a motivation for 60 studies—these studies all synthesized CTs from MRIs. Fourteen studies were motivated by synthesizing unobtained scans to aid diagnosis. Eight studies were motivated by increasing the size of paired datasets by synthesizing missing modalities.

### 3.5. Deep Learning Used

**Figure 6:**
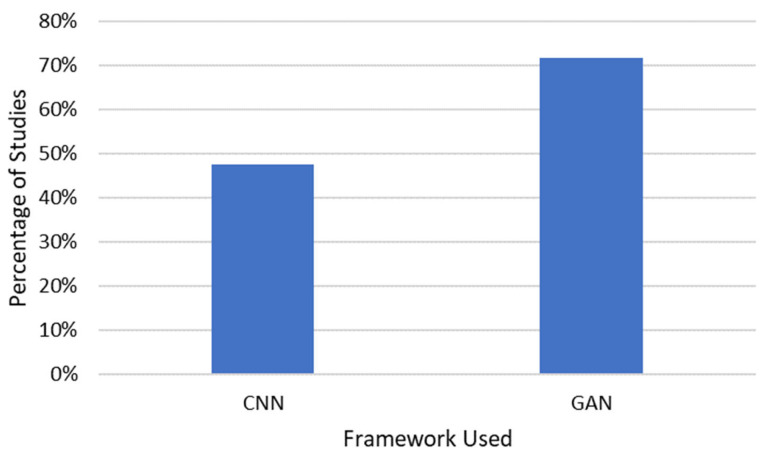
Deep learning frameworks used for medical image synthesis.

GANs were the main type of deep learning algorithm used, with 72% of studies incorporating a GAN and 48% studies incorporating a CNN (Figure 6).

### 3.6. Dataset Sizes

**Figure 7:**
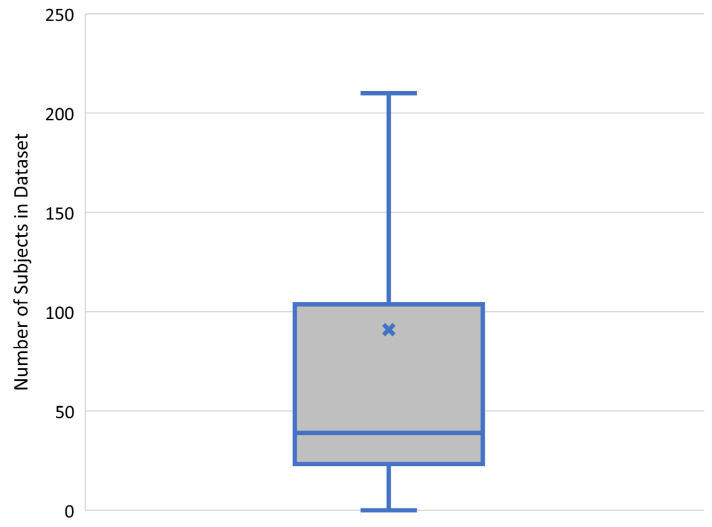
Boxplot of number of patients comprising dataset (axis limited to exclude extremes). Blue X marks the mean.

The number of subjects in the dataset had a mean of 91 and median of 39 (Figure 7). Some of the studies with smaller datasets used the leave-one-out method where the model is trained on all the data but one instance and then tested on the one instance that is left out. This is then repeated, leaving each piece of data out in turn. The mean number of patient in the dataset for cross-MRI synthesis was 274, much larger than the means for MRI to CT (56) and CT to MRI (134).

## 4. Discussion

This systematic review analyzed the current state of medical image synthesis using deep learning. The year of publication; type of synthesis; machine learning framework; dataset size; motivation; and evaluation methods used were analyzed.

The most common synthesis was MRI to CT synthesis, and almost every study performing this synthesis was motivated by MRI-only radiation therapy. The benefits of MRI-only radiotherapy are that the patient does not have to be exposed to the radiation of the CT scan, and that time and money are saved. Other motivations included turning datasets of MRIs into paired MRI/sCT datasets and completing datasets by synthesizing missing CTs. Minimal research has been conducted on MRI synthesis from CT scans. Since CTs are often the first or only scans taken for neurological issues, the time advantage and additional information from CT-synthesized MRI would be clinically beneficial. MRI gives superior tissue contrast for the diagnosis of several brain diseases and disorders, such as stroke and traumatic brain injury. CT-synthesized MRI could improve the speed and quality of treatment for stroke patients and provide a solution for the cross-modality registration problem in the context of comparing patients’ CT scans to MRI brain atlases. Depending on the training dataset, the generation of T1, T2 weighted, or even FLAIR images from CT could be investigated. These different types of MR modalities provide complementary information which can be utilized for diagnostic purposes and for registration to different brain atlases. Eleven papers [18,19,20,21,22,23,24,25,26,27,28] studied MRI synthesis from CT which demonstrates a knowledge gap in this area.

The lack of paired MRI/CT datasets is a significant problem that inhibits the use of supervised learning for cross-modality synthesis. It is therefore suggested that future studies investigate whether within-modality synthesis models could be used to generate paired datasets. Paired MRI/CT datasets are useful for a variety of applications, including training models for cross-modality synthesis and training models to perform other tasks that require paired data.

In part due to the lack of consensus on which metrics to use for evaluation, there does not appear to be a consensus on the level of accuracy required for synthetic medical images. The quality of the generated images in some publications is an area of particular concern, as some models output blurry images which mask the details of smaller-scale features. A benchmark image quality for the models for use in a clinical setting is much needed. This task will be hampered, however, by different motivations, since different studies may require different levels of accuracy and image quality. Research that helps provide a consensus or that gives guidance on the best evaluation methods is warranted to improve the progress towards clinically useful synthesized medical images.

There were a range of research motivations across the different studies; however, most papers did not mention more than one of these. The motivations for MRI synthesis from CT were quite different to the motivations for CT synthesis from MRI. A focus for future research should be establishing how different motivations for medical image synthesis affect how the synthesized images should be assessed and evaluated. This would help establish which methods perform best for medical image generation in different contexts. The motivations of the studies strongly affected the methods of evaluation used. A common evaluation method for the CTs generated from MRI for the purpose of MRI-only radiotherapy was dosimetric evaluation, which does not make sense for other types of synthesis. Research investigating clinical uses for synthetic medical images would therefore be significant.

The studies reviewed did not provide much insight into how different machine learning frameworks compare for medical image translation. The research has instead been focused on demonstrating that synthesizing medical images with deep learning is feasible. Studies used GANs and CNNs, but no particular focus was put on finding out which of these frameworks is more suited to the problem. Many of the papers used GANs, and a selection of these introduced novel contributions to the GAN model that they implemented to improve image synthesis. A much smaller selection of the papers used CNNs, and most of these did not implement novel features to adapt the models for this type of synthesis. It is recommended research be carried out on how CNNs can be adapted for this type of synthesis.

GANs are renowned for image generation, and this is presumably why they have been used so often in this area. The reason they are so popular for image generation is because they produce high-quality images due to matching the training distribution. With a dataset of medical images, the distribution statistics will be affected by the percentage of scans with artifacts such as lesions. This leads to the possibility of hallucinating or erasing lesions or other artifacts. Even in the case of supervised models such as Pix2Pix, the models still fit to the distribution of the training data [104]. CNNs only fit to the one-to-one pairings between the paired data input. This means they require a lot more data than GANs for stable training, however, this ensures the model learns the relationship between the input and output modalities. The papers including CNNs mostly used UNet and variations of UNet. Despite UNet being normally used for segmentation, a model of this architecture has proved to work well for image synthesis. A few papers did compare GANs against CNNs, however, no consistent consensus on their relative performance was found.

More studies are required to determine which deep learning architectures and implementations work best for medical image synthesis. To assist the development of this area, it is recommended that future research test and compare different methods of evaluating synthesized medical images, in order to determine the level of accuracies required for the synthesized images to be clinically useful in different contexts. Finally, it is recommended that the feasibility of a model generating pairs of synthetic CTs and synthetic MRIs be investigated. This has not been previously done and would have helpful implications for using deep learning for synthesis, segmentation, and a variety of other clinical tasks if feasible. Lack of available large medical datasets is an ongoing issue; it is therefore recommended that a global consortium be established to collate currently available datasets and coordinate with researchers and medical professionals to encourage ongoing collaboration.

## 5. Conclusions

In conclusion, this systematic review has revealed a knowledge gap within the field of medical image synthesis. Specifically, very limited research has been conducted on synthesizing MRIs from CT scans, despite a variety of motivations. Since MRIs give superior tissue contrast and are preferred for the diagnosis of several brain diseases and disorders, synthesis of such data from CTs (which are more commonly obtained) would be clinically beneficial. All studies reviewed on medical image translation have been published since 2017, making this a relatively new area—as such, there is little consensus around methods of assessing and testing the performance of models for this task. We therefore recommend that more research be conducted into MRI synthesis from CT scans. Current advances in deep learning have shown clinical utility for stroke and traumatic brain injury patients, making this approach promising as a candidate for solving the cross-modality registration problem. Recommendations were given for the directions of future research in this field, including on a related application (not yet discussed in the literature) of using image synthesis techniques to generate pairwise datasets. It was concluded that more research is required to determine which deep learning methods are most effective and accurate in synthesizing medical images for use in a clinical setting.

## Figures and Tables

**Table 1:** Search terms used for the electronic databases.

Category	Search Terms
Machine Learning	machine learning, GAN, generative adversarial network, convolutional neural network, artificial intelligence, deep learning
Image Generation	synth*, generat*, pseudo*, transform*
Medical Imaging	MRI, MR, CT, PET

**Table 2:** Descriptions of the Synthesis Type and Motivations categories.

Extracted Variable	Categories	Description
Synthesis Type	CT to MRI	Using a CT to generate an MRI
	MRI to CT	Using an MRI to generate a CT
	Cross MRI	Using one MRI sequence to generate a different MRI modality. For example, using a T1w MRI to generate a T2w MRI.
	MRI to PET	Using an MRI to generate a PET
	PET to CT	Using a PET to generate a CT
Motivations	Aid Diagnosis	Synthesizing unobtained scans to provide extra information for diagnosis
	Missing Data	Improving paired datasets by synthesizing missing scans
	Memory Efficiency	Improving the memory efficiency of synthesis models so that high quality scans can be synthesized
	Attenuation Correction	Synthesizing scans of a modality which can aid in attenuation correction of PETs
	Multimodal Registration	Synthesizing scans of a modality which is simpler to register to the target.
	MRI-only Radiation Therapy	Synthesizing a CT so that a patient only requires an MRI before radiation therapy
	Reduce Radiation	Synthesizing a scan which would otherwise expose the patient to radiation
	Segmentation	Synthesizing scans of a modality which can help segmentation models either in training or in segmenting the scan

**Table 3:** A summary of the data extracted from the reviewed literature.

Paper	Year	Synthesis Type	Motivations	Body Part	Model Framework	Number of Patients	Evaluation Methods
[5]	2020	Cross MRI	Aid Diagnosis	Brain	GAN	274	SSIM, PSNR, NMSE
[6]	2022	Cross MRI	Aid Diagnosis	Brain	GAN, CNN	Unspecified	SSIM, MSE, PSNR, VIF, FID
[7]	2020	Cross MRI	Aid Diagnosis	Brain	CNN	15	PSNR, SSIM, HFEN
[8]	2022	Cross MRI	Aid Diagnosis	Brain	CNN	Unspecified	MAPE, RSMPE, SSIM
[9]	2020	Cross MRI	Increase Data	Brain	GAN	1113	Estimated Divergence
[10]	2020	Cross MRI	Memory Efficiency	Brain	GAN	274	SSIM, MAE, PSNR, MSE
[11]	2022	Cross MRI	Aid Diagnosis, Increase Data	Brain	GAN	127	MAE, SSIM, PSNR, MI
[12]	2019	Cross MRI	Aid Diagnosis	Brain	CNN	15	SSIM
[13]	2023	Cross MRI	Aid Diagnosis	Brain	GAN	128	MAE, SSIM, PSNR
[14]	2022	Cross MRI	Segmentation	Brain	GAN	210	DSC, ASSD
[15]	2022	Cross MRI	Aid Diagnosis	Brain	GAN	285	SSIM, PSNR, Experts
[16]	2023	Cross MRI	Increase Data	Brain	GAN	372	MSE, SSIM
[17]	2021	Cross MRI	Increase Data	Brain	GAN	199	MSE, SSIM, PSNR
[18]	2022	CT to MRI	Aid Diagnosis	Lumbar	GAN	285	SSIM, PSNR, Experts
[19]	2020	CT to MRI	Increase Data	Brain	GAN, CNN	34	MAE, SSIM, PSNR
[20]	2021	CT to MRI	Increase Data	Pelvis	GAN, CNN	17	PSNR, SSIM, Experts, DSC
[21]	2021	CT to MRI	Increase Data	Head and Neck	GAN	202	Segmentation
[22]	2020	CT to MRI	Multimodal Registration, Aid Diagnosis	Brain	GAN, CNN	34	MAE, MSE, SSIM, PSNR
[23]	2019	CT to MRI	Segmentation	Pelvis	GAN	140	Segmentation
[24]	2021	CT to MRI	Segmentation	Head and Neck	GAN	118	Segmentation
[25]	2023	CT to MRI	Aid Diagnosis, Multimodal Registration	Brain	GAN, CNN	181	MAE, MSE, PSNR, SSIM, Registration, DSC
[26]	2019	CT to MRI	Segmentation, Aid Diagnosis	Brain	GAN	94	DSC, HD
[27]	2022	CT to MRI	Aid Diagnosis	Brain	GAN	103	Experts
[28]	2021	CT to MRI, MRI to CT	Aid Diagnosis	Prostate	GAN	271	KID, FID, DSC
[29]	2022	MRI to CT	Attenuation Correction	Whole Body	CNN	46	MAE, Regional Analysis, Correlation
[30]	2021	MRI to CT	Aid Diagnosis	Lumbar	CNN	30	Regional Analysis
[31]	2022	MRI to CT	Aid Diagnosis	Hip	CNN	27	Regional Analysis
[32]	2021	MRI to CT	Aid Diagnosis	Sacroiliac Joint	CNN	30	Diagnostic Accuracy
[33]	2022	MRI to CT	Aid Diagnosis	Hip	CNN	30	Regional Analysis
[34]	2023	MRI to CT	Aid Diagnosis	Knee	CNN	69	Diagnostic Accuracy
[35]	2023	MRI to CT	MRI-only Radiation Therapy	Brain	GAN, CNN	104	MAE, Dosimetric
[36]	2020	MRI to CT	MRI-only Radiation Therapy	Brain	GAN	77	MAE
[37]	2022	MRI to CT	MRI-only Radiation Therapy	Head and Neck	CNN	47	MAE, SSIM, Dosimetric
[38]	2020	MRI to CT	MRI-only Radiation Therapy	Brain	GAN	60	MAE, Dosimetric
[39]	2022	MRI to CT	MRI-only Radiation Therapy	Head and Neck	GAN	206	MAE, Dosimetric
[40]	2019	MRI to CT	MRI-only Radiation Therapy	Liver	GAN	21	MAE, Dosimetric
[41]	2021	MRI to CT	MRI-only Radiation Therapy	Head and Neck	GAN	56	MAE, SSIM, PCC, FID, SWD, BD, PSNR, DSC
[42]	2020	MRI to CT	MRI-only Radiation Therapy	Pelvis	CNN	15	ME, MAE, SSIM, PSNR, PCC
[43]	2021	MRI to CT	MRI-only Radiation Therapy	Pelvis	GAN, CNN	20	ME, MAE, PCC, SSIM, PSNR
[44]	2021	MRI to CT	MRI-only Radiation Therapy	Head and Neck	GAN, CNN	164	MAE, ME, PSNR
[45]	2021	MRI to CT	MRI-only Radiation Therapy	Prostate	GAN	113	ME, MAE, PSNR
[46]	2021	MRI to CT	MRI-only Radiation Therapy	Brain	GAN, CNN	18	MAE, MSE, PSNR, SSIM, PCC
[47]	2019	MRI to CT	MRI-only Radiation Therapy	Liver	GAN, CNN	21	NCC, MAE, PSNR
[48]	2019	MRI to CT	MRI-only Radiation Therapy	Brain	GAN	77	MAE, DSC
[49]	2021	MRI to CT	MRI-only Radiation Therapy	Head and Neck	CNN	23	MAE, Dosimetric
[50]	2023	MRI to CT	MRI-only Radiation Therapy	Abdomen	GAN, CNN	76	Dosimetric
[51]	2019	MRI to CT	MRI-only Radiation Therapy	Head and Neck	CNN	34	MAE, ME, Dosimetric
[52]	2021	MRI to CT	MRI-only Radiation Therapy	Brain	GAN	37	Dosimetric
[53]	2020	MRI to CT	MRI-only Radiation Therapy	Pelvis	GAN	120	Dosimetric
[54]	2019	MRI to CT	MRI-only Radiation Therapy	Brain	CNN	60	MAE
[55]	2023	MRI to CT	MRI-only Radiation Therapy	Abdomen	CNN	39	MAE, Dosimetric
[56]	2022	MRI to CT	MRI-only Radiation Therapy	Prostate	GAN	39	MAE, ME, MAPE, DSC
[57]	2020	MRI to CT	MRI-only Radiation Therapy	Abdomen	GAN	12	MAE, Dosimetric
[58]	2022	MRI to CT	MRI-only Radiation Therapy	Thorax	GAN	60	MAE, ME, Dosimetric
[59]	2022	MRI to CT	MRI-only Radiation Therapy	Brain	GAN	24	MAE, PSNR, SSIM
[60]	2021	MRI to CT	MRI-only Radiation Therapy	Brain	CNN	30	ME, MAE, MSE
[61]	2021	MRI to CT	MRI-only Radiation Therapy	Pelvis	GAN	38	MAE, Dosimetric
[62]	2020	MRI to CT	MRI-only Radiation Therapy	Pelvis	GAN	19	MAE
[63]	2020	MRI to CT	MRI-only Radiation Therapy	Abdomen	CNN	31	MAE, Dosimetric
[64]	2021	MRI to CT	MRI-only Radiation Therapy	Head and Neck	CNN, GAN	35	MAE, SSIM, PSNR
[65]	2022	MRI to CT	MRI-only Radiation Therapy	Pelvis	GAN	40	Dosimetric
[66]	2021	MRI to CT	MRI-only Radiation Therapy	Brain	CNN	20	MAE, Dosimetric
[67]	2022	MRI to CT	MRI-only Radiation Therapy	Brain	CNN	21	Dosimetric
[68]	2018	MRI to CT	MRI-only Radiation Therapy	Pelvis	GAN	91	Dosimetric
[69]	2020	MRI to CT	MRI-only Radiation Therapy	Head and Neck	GAN, CNN	45	MAE, SSIM, PSNR, DSC, Dosimetric
[70]	2020	MRI to CT	MRI-only Radiation Therapy	Pelvis	CNN	23	MAE, ME, DSC, Regional Analysis, PSNR
[71]	2021	MRI to CT	MRI-only Radiation Therapy	Prostate	CNN	30	MAE
[72]	2019	MRI to CT	MRI-only Radiation Therapy	Thorax	GAN	60	RMSE, SSIM, PSNR, Dosimetric
[73]	2022	MRI to CT	MRI-only Radiation Therapy	Prostate	GAN	57	MAE, PSNR, SSIM, Dosimetric
[74]	2022	MRI to CT	MRI-only Radiation Therapy	Brain	GAN	54	MAE, SSIM, Dosimetric
[75]	2021	MRI to CT	MRI-only Radiation Therapy	Pelvis	GAN, CNN	30	MAE, RMSE, PSNR, SSIM
[76]	2021	MRI to CT	MRI-only Radiation Therapy	Brain	GAN	184	Dosimetric
[77]	2017	MRI to CT	MRI-only Radiation Therapy	Brain	CNN	18	MAE, MSE, PCC
[78]	2021	MRI to CT	MRI-only Radiation Therapy	Brain	GAN	12	Dosimetric, Registration
[79]	2019	MRI to CT	MRI-only Radiation Therapy	Brain	GAN	24	MAE, PSNR, NCC
[80]	2019	MRI to CT	MRI-only Radiation Therapy	Prostate	GAN	17	MAE, Dosimetric
[81]	2020	MRI to CT	MRI-only Radiation Therapy	Head and Neck	GAN	173	MAE, Dosimetric
[82]	2019	MRI to CT	MRI-only Radiation Therapy	Head and Neck	CNN	33	MAE, ME
[83]	2023	MRI to CT	MRI-only Radiation Therapy	Head and Neck	GAN	79	MAE, PSNR, SSIM
[75]	2021	MRI to CT	MRI-only Radiation Therapy	Thorax	GAN, CNN	30	MAE, RMSE, PSNR, SSIM
[75]	2021	MRI to CT	MRI-only Radiation Therapy	Abdomen	GAN, CNN	30	MAE, RMSE, PSNR, SSIM
[79]	2019	MRI to CT	MRI-only Radiation Therapy	Pelvis	GAN	20	MAE, PSNR, NCC
[19]	2020	MRI to CT	MRI-only Radiation Therapy	Brain	GAN, CNN	34	MAE, SSIM, PSNR
[84]	2021	MRI to CT	Multimodal Registration	Head and Neck	GAN	25	Registration
[85]	2021	MRI to CT	Reduce Radiation	Lower Arm	GAN	8	Surgical Planning Errors
[86]	2022	MRI to CT	Reduce Radiation	Head and Neck	CNN	39	MAE, MSE
[87]	2020	MRI to CT	Multimodal Registration	Head and Neck	GAN, CNN	9	MAE, PCC, SLPD
[88]	2022	MRI to CT	Segmentation	Abdomen	GAN	34	Segmentation
[89]	2018	MRI to CT	Attenuation Correction	Brain	CNN	7	PSNR, Correlation
[90]	2020	MRI to CT	MRI-only Radiation Therapy	Brain	GAN	15	MAE
[91]	2022	MRI to CT	Aid Diagnosis	Pelvis	GAN, CNN	19	SSIM
[92]	2018	MRI to CT	Attenuation Correction	Brain	CNN	20	MAE, PET Reconstruction
[93]	2017	MRI to CT	MRI-only Radiation Therapy	Brain	GAN	24	MAE, PSNR
[94]	2018	MRI to CT	MRI-only Radiation Therapy	Brain	GAN	45	MAE, PSNR, SSIM
[95]	2021	MRI to CT	MRI-only Radiation Therapy	Brain	GAN	45	MAE, PSNR, SSIM
[96]	2021	MRI to CT	MRI-only Radiation Therapy	Abdomen	GAN	89	MAE, DSC
[97]	2023	MRI to CT	MRI-only Radiation Therapy	Brain	GAN	95	MAE, GPR
[98]	2019	MRI to CT	Attenuation Correction	Brain	CNN	400	MAE, PET Reconstruction
[99]	2021	MRI to CT	MRI-only Radiation Therapy	Brain	GAN, CNN	86	MAE, SSIM, PSNR
[100]	2021	MRI to PET	Increase Data	Whole Body	CNN	56	AC
[101]	2022	MRI to PET	Aid Diagnosis	Brain	CNN	120	PSNR, SSIM
[102]	2021	MRI to PET	Aid Diagnosis	Brain	GAN	481	MAE, SSIM, PSNR
[103]	2022	PET to CT	Reduce Radiation, Attenuation Correction	Whole Body	GAN	34	NRMSE, PSNR, PCC, SSIM

## Abbreviations

MAE	Mean absolute error
PSNR	Peak signal-to-noise ratio
SSIM	Structural similarity index measure
ME	Mean error
MSE	Mean squared error
DSC	Dice score
PCC	Pearson correlation coefficient
FID	Fréchet inception distance
RMSE	Root mean squared error
NCC	Normalized cross correlation
MAPE	Mean absolute percentage error
VIF	Visual information fidelity
BD	Bjøntegaard-Delta
HD	Hausdorff distance
HFEN	High-frequency error norm
MI	Mutual information
NRMSE	Normalized root mean squared error
RSMPE	Root mean squared percentage error
SD	Sharpness difference
SLPD	Sum of local phase differences
SWD	Sliced Wasserstein discrepancy
NMSE	Normalized mean squared error
